# Genetic characterization of clinical and agri-food isolates of multi drug resistant *Salmonella enterica *serovar Heidelberg from Canada

**DOI:** 10.1186/1471-2180-8-89

**Published:** 2008-06-06

**Authors:** Ashleigh K Andrysiak, Adam B Olson, Dobryan M Tracz, Kathryn Dore, Rebecca Irwin, Lai-King Ng, Matthew W Gilmour, Canadian Integrated Program for Antimicrobial Resistance Surveillance Collaborative

**Affiliations:** 1 Bacteriology and Enteric Diseases Program, National Microbiology Laboratory, Public Health Agency of Canada, Winnipeg, MB, Canada; 2 Department of Medical Microbiology, University of Manitoba, Winnipeg, MB, Canada; 3 Foodborne, Waterborne and Zoonotic Infections Division, Public Health Agency of Canada, Guelph, ON, Canada; 4 Laboratory for Foodborne Zoonoses (LFZ), Public Health Agency of Canada, St-Hyacinthe, QC and Guelph, ON, Canada

## Abstract

**Background:**

*Salmonella enterica *serovar Heidelberg ranks amongst the most prevalent causes of human salmonellosis in Canada and an increase in resistance to extended spectrum cephalosporins (ESC) has been observed by the Canadian Integrated Program for Antimicrobial Resistance Surveillance. This study examined the genetic relationship between *S*. Heidelberg isolates from livestock, abattoir, retail meat, and clinical human specimens to determine whether there was a link between the emergence of MDR *S*. Heidelberg in chicken agri-food sources and the simultaneous increase of MDR *S*. Heidelberg in human clinical samples.

**Results:**

Chromosomal genetic homogeneity was observed by pulsed-field gel electrophoresis (PFGE), DNA sequence-based typing (SBT) and DNA microarray-based comparative genomic hybridization (CGH). Sixty one percent of isolates were indistinguishable by PFGE conducted using *Xba*I and *Bln*I restriction enzymes. An additional 15% of isolates had PFGE patterns that were closely related to the main cluster. SBT did not identify DNA polymorphisms and CGH revealed only genetic differences between the reference *S*. Typhimurium strain and *S*. Heidelberg isolates. Genetic variation observed by CGH between *S*. Heidelberg isolates could be attributed to experimental variation. Alternatively, plasmid content was responsible for differences in antimicrobial susceptibility, and restriction fragment length polymorphism (RFLP) analyses followed by replicon typing identified two divergent plasmid types responsible for ESC resistance.

**Conclusion:**

Due to the overall limited genetic diversity among the isolates, it was not possible to identify variable traits that would be suitable for source tracking between human and agri-food isolates of *S*. Heidelberg in Canada.

## Background

Non-typhoidal serovars of *Salmonella enterica *subspecies *enterica *are responsible for outbreaks and sporadic occurrences of gastrointestinal illness that result in significant human morbidity worldwide [1]. Human salmonellosis in North America is attributed primarily to *S*. *enterica *serovars Typhimurium and Enteritidis, with other serovars that vary in regional prevalence constituting the remainder [2,3]. Due to the high incidence of *S*. Typhimurium and *S*. Enteritidis, serotyping alone is not sufficient to establish the chain of transmission from putative animal, environmental or food sources to human cases. Serotyping in combination with other more discriminatory methods such as phage typing, pulsed-field gel electrophoresis (PFGE), DNA sequence-based typing (SBT), comparative genomic hybridizations (CGH) or plasmid profiling may provide useful subtyping information. For example, serotyping has been used in combination with PFGE to successfully link *S. enterica *isolates recovered from agri-food products to outbreak-associated cases of human salmonellosis [4,5].

*S*. Heidelberg is a member of *Salmonella enterica *subspecies *enterica *serogroup B and is highly prevalent in both Canada and the United States, but is infrequently reported in European countries [3,6]. In Canada, *S*. Heidelberg consistently ranks with *S*. Enteriditis and *S*. Typhimurium as one of the top three most prevalent causes of human salmonellosis, accounting for between 12–20% of human clinical *Salmonella *isolates reported annually between 2000 and 2005 [2]. *S*. Heidelberg is also one of the most common *Salmonella *serovars isolated from broiler chickens and egg laying flocks in Canada and accordingly outbreaks of human *S*. Heidelberg infections have been associated with consumption of contaminated poultry products including chicken, chicken nuggets and eggs [7,8]. Other modes of transmission such as person-to-person spread or direct contact with infected animals have rarely been reported [7].

The Canadian Integrated Program for Antimicrobial Resistance Surveillance was established by the Public Health Agency of Canada (PHAC) in 2002. CIPARS collects information regarding antimicrobial resistance trends amongst enteric pathogens isolated from human clinical specimens, livestock and retail meat sources to assess the human health risk associated with the use of antimicrobials in food animal production. CIPARS has noted that *S*. Heidelberg was frequently isolated from poultry sources, with this serovar accounting for 72% of all *Salmonella *isolates recovered from retail chicken in 2004 [8]. In addition, there has been an increase in resistance to multiple antibiotics, particularly extended spectrum cephalosporins (ESC), amongst S. Heidelberg isolates from human and agri-food sources [2,8].

In this study, the genetic relationship between *S*. Heidelberg isolates from retail chicken, abattoir chicken ceca and chicken, cattle, swine and human clinical samples was examined using a variety of molecular methods. Our goal was to identify variable genetic traits that would support the investigation of potential linkages between human multi-drug resistant (MDR) *S*. Heidelberg isolates and MDR *S*. Heidelberg in chicken agri-food sources.

## Results and Discussion

### Genetic variation due to chromosomal determinants

**Figure 1:**
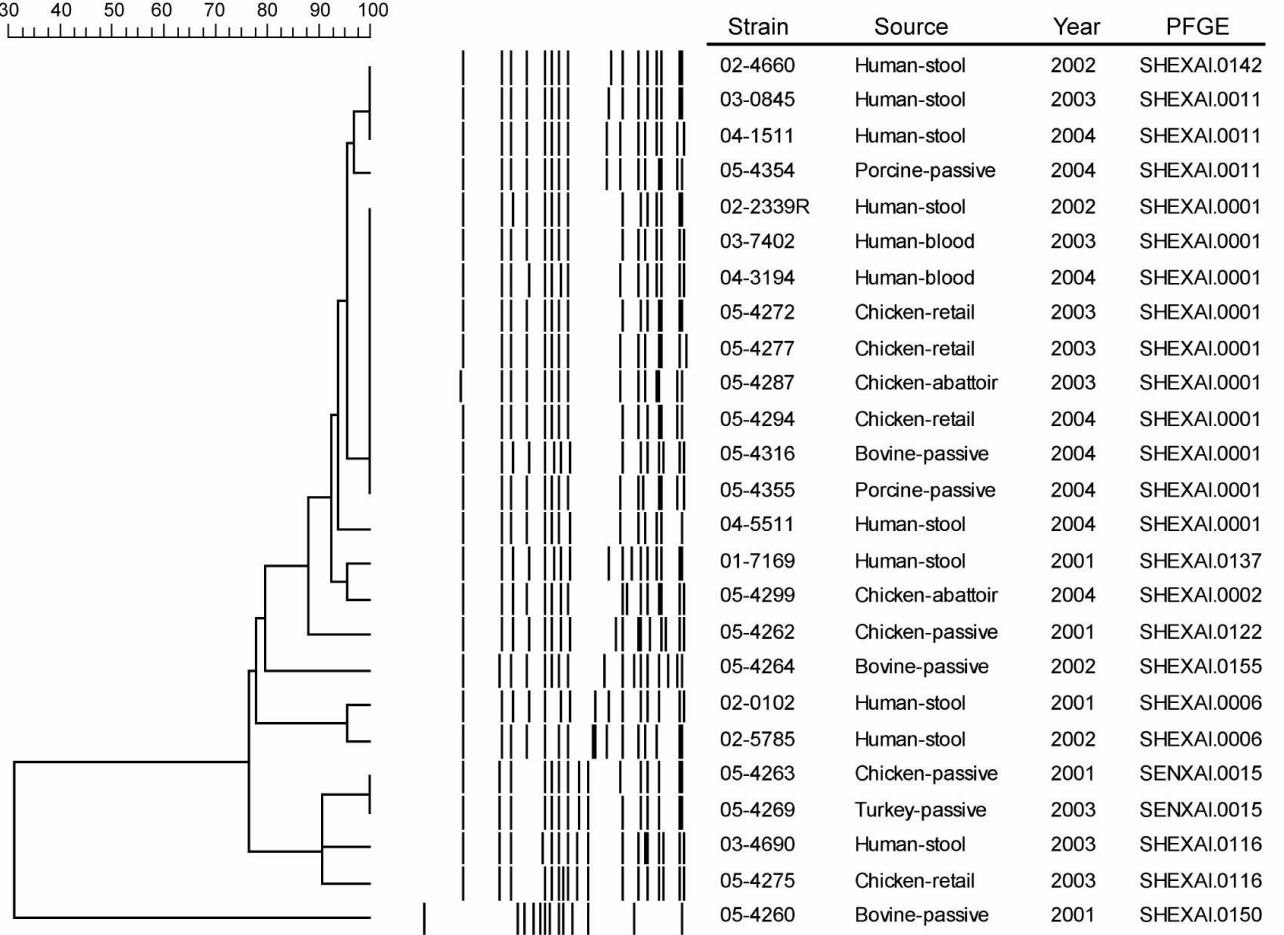
**Dendrogram of *S*. Heidelberg DNA macrorestriction patterns generated using *Xba*I.** Strains were selected to present a diverse range of patterns. Dendrogram was created using Applied Maths Bionumerics version 4.0 using unweighted pair group method (UPGMA) with a dice coefficient of similarity, 1% band tolerance and 1.5% optimization. The scale bar indicates percent similarity.

Pulsed-field gel electrophoresis (PFGE) is frequently used to subtype *Salmonella *[3,9] and has previously been used to link cases of human salmonellosis with contaminated agri-food products [4]. A comparison of PFGE data for all 107 *S*. Heidelberg isolates submitted by CIPARS to PulseNet Canada from 2001 to 2004 for PFGE analyses was retrospectively performed. PFGE identified limited genetic diversity amongst these human and non-human isolates originating from 5 provinces, as 61% of isolates were indistinguishable by *Xba*I and *Bln*I PFGE (patterns SHEXAI.0001 and SHEBNI.0001, respectively; data not shown). An additional 15% of the isolates differed from pattern SHEXAI.0001 by 1–3 fragments and therefore meet the criteria of being closely related to this restriction pattern [10]. The lack of genetic diversity observed using PFGE therefore makes this method unsuitable for identification of epidemiological links between human and agri-food isolates. Thus a subset of up to 39 strains (Table 1; Figure 1) representing a diverse range of temporally and geographically distinct *S*. Heidelberg isolates with different PFGE types, sources, sites of isolation, phage types and resistance phenotypes were selected for examination by additional subtyping methods.

**Table 1:** Bacterial strains used in this study and PCR-based replicon typing and resistance gene characterization for *S*. Heidelberg and *E. coli bla*_cmy-2 _plasmids.

Strain^a^	Year	PT^b^	Province^c^	Source	Resistance Phenotype^d^	Resistance Genotype^e^	*bla*_cmy-2 _plasmid mediated resistance determinants^e^	Rep^h^
01–7169	2001	29	ON	Human stool	Amp, Chl, Cro, Gen, Kan, Str, Sxt, Tio	*aadA2, strA, sul1*	n/a	n/a
02–0102	2001	11a	ON	Human stool	Chl, Str, Sul	*aadA1, aadA2*	n/a	n/a
02–2339^fg^	2002	19	ON	Human stool	Sensitive	n/a	n/a	n/a
02–4660^fg^	2002	19	ON	Human stool	Amc, Amp, Chl, Fox, Str, Sxt, Tet, Tio	*bla*_cmy-2_, *dhfrA1, floR, strA, sul1, tetA*	*bla*_cmy-2_, *dhfRA1, floR, strA, sul1, tetA*	A/C
02–5785^f^	2002	AT02–5785	ON	Human stool	Amc, Amp, Chl, Fox, Gen, Kan, Smx, Str, Tet, Tio	*bla*_cmy-2_, *floR, strA, sul1, tetA*	n/i	n/a
02–5980	2002	29	QC	Human blood	Amc, Amp, Fox, Tio	*bla*_cmy-2_	*bla*_cmy-2_	I1
03–0262^fg^	2003	41	QC	Human blood	Amc, Amp, Fox, Cep, Cro, Tio	*bla*_cmy-2_	*bla*_cmy-2_	I1
03–0845^f^	2003	29	ON	Human stool	Amc, Amp, Fox, Cep, Chl, Gen, Kan, Smx, Str, Tet, Tio	*aadB, bla*_cmy-2_, *cmlA*, *strA, sul1, tetA*	*aadB, bla*_cmy-2_,*cmlA, strA, sul1, tetA*	A/C
03–3012	2003	4	QC	Human blood	Amc, Amp, Cep, Cro, Fox, Tio	*bla*_cmy-2_	bla_cmy-2_	I1
03–4601	2003	AT03–4601	QC	Human blood	Amc, Amp, Cep, Chl, Cro, Fox, Tio, Smx, Str, Tet, Tio	*bla*_cmy-2_, *floR, strA, tetA*	*bla*_cmy-2_, *floR*, *strA*, *tetA*	A/C; I1
03–4690	2003	32	ON	Human stool	Amc, Amp, Cep, Cro, Fox, Gen, Str, Tet, Tio	*bla*_cmy-2_, *strA, tetB*	*bla*_cmy-2_	I1
03–7402^fg^	2003	29	ON	Human blood	Amc, Amp, Cep, Cro, Fox, Tio	*bla*_cmy-2_	*bla*_cmy-2_	I1
04–0346^fg^	2004	54	QC	Human stool	Amc, Amp, Cep, Chl, Cro, Fox, Smx, Str, Tet, Tio	*bla*_cmy-2_, *floR, strA, tetA*	*bla*_cmy-2_, *floR, tetA*	A/C
04–1511	2004	41	ON	Human stool	Amc, Amp, Cep, Fox, Smx, Str, Tet, Tio	*bla*_cmy-2_, *strA, tetA*	*bla*_cmy-2_	I1
04–3194	2004	29	ON	Human blood	Amc, Amp, Cro, Fox, Tio	*bla*_cmy-2_	*bla*_cmy-2_	I1
04–3293	2004	29a	QC	Human blood	Amc, Amp, Cro, Fox, Tio	*bla*_cmy-2_	*bla*_cmy-2_	I1
04–4717	2004	29	QC	Human blood	Amc, Amp, Cro, Fox, Tio	*bla*_cmy-2_	*bla*_cmy-2_	I1
05–5435	2004	29	QC	Human blood	Amc, Amp, Cro, Fox, Tet, Tio	*bla*_cmy-2_	*bla*_cmy-2_	I1
04–5511^fg^	2004	41	ON	Human stool	Sensitive	n/a	n/a	n/a
05–4260	2001	29	ON	Bovine passive	Amc, Amp, Cep, Fox, Tio	n/d	n/a	n/a
05–4262^fg^	2001	Untypable	AB	Chicken passive	Amc, Amp, Cep, Chl, Fox, Gen, Kan, Smx, Str, Tet, Tio	*bla*_cmy-2_, *floR, strA, sul1, tetA*	*bla*_cmy-2_, *floR, strA, sul1, tetA*	A/C; I1
05–4263^g^	2001	Untypable	ON	Chicken passive	Amc, Amp, Cep, Fox, Tet, Tio	*bla*_cmy-2_, *tetB*	*bla*_cmy-2_	I1
05–4264	2002	Atypical	ON	Bovine passive	Amc, Amp, Cep, Chl, Fox, Gen, Kan, Str, Sxt, Tet, Tio	*aadA1, bla*_cmy-2_, *dhfRA1, floR, strA, sul1, tetA*	n/i	n/a
05–4269^fg^	2003	29	ON	Turkey passive	Amc, Amp, Cep, Fox, Tio	*bla*_cmy-2_	*bla*_cmy-2_	I1
05–4272	2003	29	QC	Chicken retail	Amc, Amp, Cep, Fox, Tio	*bla*_cmy-2_	*bla*_cmy-2_	I1
05–4275	2003	32	QC	Chicken retail	Amc, Amp, Cep, Fox, Gen, Str, Tet, Tio	*bla*_cmy-2_, *strA, tetB*	*bla*_cmy-2_	I1
05–4277	2003	29	QC	Chicken retail	Amc, Amp, Cep, Fox, Tio	*bla*_cmy-2_	*bla*_cmy-2_	I1
05–4287	2003	29	ON	Chicken abattoir	Amc, Amp, Cep, Fox, Tio	*bla*_cmy-2_	*bla*_cmy-2_	I1
05–4294	2004	29	ON	Chicken retail	Amc, Amp, Cep, Fox, Tio	*bla*_cmy-2_	*bla*_cmy-2_	I1
05–4299	2004	29	ON	Chicken abattoir	Amc, Amp, Cep, Fox, Tio	*bla*_cmy-2_	*bla*_cmy-2_	Unknown
05–4316^f^	2004	29	PE	Bovine passive	Amc, Amp, Cep, Fox, Tio	*bla*_cmy-2_	*bla*_cmy-2_	I1
05–4354^fg^	2004	41	QC	Porcine passive	Amc, Amp, Cep, Fox, Tio	*bla*_cmy-2_	*bla*_cmy-2_	Unknown
05–4355	2004	29	QC	Porcine passive	Amc, Amp, Cep, Fox, Tio	*bla*_cmy-2_	*bla*_cmy-2_	I1
00–5440	2000	29	AB	Human	Sensitive	n/a	n/a	n/a
05–1147	2005	29	QC	Human	Sensitive	n/a	n/a	n/a
539	unknown	n/d	ON	Chicken rinse	Sensitive	n/a	n/a	n/a
564	unknown	n/d	ON	Egg yolk mix	Sensitive	n/a	n/a	n/a
1170	unknown	n/d	ON	Cocoa beans	Sensitive	n/a	n/a	n/a
S-467	1948	n/d	BC	Human	Sensitive	n/a	n/a	n/a
*E. coli *830	2004	n/d	AB	Chicken abattoir	Amc, Amp, Cep, Fox, Gen, Smx, Str, Tet, Tio	*aadA1, bla*_cmy-2_, *sul1*	*aadA1, bla*_*cmy*2_	Unknown
*E. coli *831	2004	n/d	QC	Chicken abattoir	Amc, Amp, Chl, Fox, Kan, Smx, Str, Tet, Tio	*aadA1, bla*_cmy-2_, *floR, strA, sul1, tetA*	*aadA1*, bla_cmy-2_, *floR, strA, tetA*	A/C
*E. coli *832	2004	n/d	QC	Porcine abattoir	Amc, Amp, Cep, Chl, Fox, Smx, Str, Tet, Tio	*bla*_cmy-2_, *floR, strA, tetA*	bla_cmy-2_, *strA, tetA, floR*	A/C
*E. coli *833	2004	n/d	ON	Chicken abattoir	Amc, Amp, Cep, Fox, Gen, Smx, Str, Tio	*aadA1*, *bla*_cmy-2_, *floR, sul1*	*aadA1, bla*_*cmy*2_	I1
*E. coli *834	2004	n/d	QC	Bovine retail	Amc, Amp, Cep, Fox, Tio	*bla*_cmy-2_, *bla*_cmy-2_	*bla*_*cmy*2_	I1

^a ^All strains are *S*. Heidelberg unless identified otherwise

^b ^PT, phage type; n/d, not determined

^c ^AB, Alberta; BC, British Columbia; PE, Prince Edward Island; QC, Quebec; ON, Ontario

^d ^Amc, amoxicillin-clavulanic acid; Amp, ampicillin; Fox, cefoxitin; Tio, ceftiofur; Cro, cefriaxone; Cep, cephalothin; Chl, chloramphenicol; Gen, gentamycin; Kan, kanamycin; Nal, nalidixic acid; Str, streptomycin; Sul, sulfamethoxazole; Tet, tetracycline; Smx, sulfizoxazole; Sxt, trimethoprim-sulfamethoxazole. n/a, not applicable.

^e ^*aadA1*, streptomycin/spectinomycin adenyltransferase; *aadB*, aminoglycoside adenyltransferase; *cmlA*, chloramphenicol/florphenicol efflux; *dhfR*, dihydrofolate reductase; *floR*, efflux; *strA*, streptomycin phosphotransferase; *sul1*, dihydropteroate synthase; *tetA*, efflux; *tetB*, efflux; *bla*_cmy-2_, beta-lactamase. n/a, not applicable; n/i, plasmid not isolated; n/d, not determined.

^f ^CGH were conducted on these isolates

^g ^DNA sequence-based typing was performed on these isolates

^h ^n/a, not applicable

DNA sequence-based typing (SBT) was conducted on *S*. Heidelberg strains using a previously published scheme examining the *fimA, manB*, and *mdh *loci. This SBT scheme has previously been shown to distinguish amongst some *Salmonella *isolates of the same serovar, including *S*. Agona and *S*. Typhimurium [11]. Ten *S*. Heidelberg isolates were selected from human, chicken, turkey and bovine sources (including both ESC resistant and susceptible isolates, if available) for examination by SBT (Table 1; Table 2). No nucleotide polymorphisms were observed amongst 2502 bp sequenced from each of these strains therefore the application of this method offered no additional discrimination.

**Table 2:** Oligonucleotides used in this study

Primer	Target^a^	Sequence 5'-3'	Product Size (bp)	Annealing Temp	Reference
aadA1F	*aadA1*	TATCAGAGGTAGTTGGCGTCAT	484	54°C	[25]
aadA1R		GTTCCATAGCGTTAAGGTTTCATT			
aadA2F	*aadA2*	TGTTGGTTACTGTGGCCGTA	712	61°C	[25]
aadA2R		GATCTCGCCTTTCACAAAGC			
cmy-2-1	*bla*_cmy-2_	ACACTGATTGCGTCTGACG	1143	60°C	[26]
cmy-2–2		AATATCCTGGGCCTCATCG			
cmy-2–3		AGTTCTGGCCAGTATTTCG	n/a	sequencing	
cmy-2–4		TGCAACCATTAAAACTGGC	n/a	sequencing	
cmy-2–5		TTCCTTTTAATTACGGAAC	n/a	sequencing	
dhfRA1F	*dhfRA1*	GTGAAACTATCACTAATGGTAGCT	470	50°C	[27]
dhfRA1R		ACCCTTTTGCCAGATTTGGTACCT			
fimAF	*fimA*	TCAGGGAGAAACAGAAAACTAAT	760	55°C	[11]
fimAR		TCCCCGATAGCCTCTTCC			
fldA-R	*fldA*	TTAGGCGTTGAGGATGTCG	530	55°C	This study
fldA-F		GCAATCACTGGCATCTTTTTC			
floRF	*floR*	AACCCGCCCTCTGGATCAAGTCAA	548	60°C	[25]
floRR		CAAATCACCGGCCACGCTGTATC			
furR-F	*furR*	AAAGAAGGCTGGCCTGAAAG	452	50°C	This study
furR-R		TTATTTAGTCGCGTCATCGTG			
intAF	3' CS	GGCATCCAAGCAGCAAG	variable	52°C	[25]
intAR	5' CS	AAGCAGACTTGACCTGG			
intAF2a	integron	AACCTTTTTGGCCTCCAG	n/a	sequencing	This study
intAF2b		CCTCCAGCGCTTGTGTAG	n/a	sequencing	
intAF3		TCAGTGTTAGTCCCATCTCC	n/a	sequencing	
intAF4		ACTTTAGTTGGCGGTACTCC	n/a	sequencing	
intAR3		CCATAAAACGAGCCGTAAAC	n/a	sequencing	
intAR4		CAAACCTAGAAACGCAAAGA	n/a	sequencing	
manBF	*manB*	CATAACCCGATGGACTACAACG	893	55°C	[11]
manBR		ACCAGCAGCCACGGGATCAT			
mdhF	*mdh*	GATGAAAGTCGCAGTCCTCG	849	50°C	[11]
mdhR		TATCCAGCATAGCGTCCAGC			
miaB-R	*miaB*	TAGAATCCTACGCCCAGCTC	1424	55°C	This study
miaB-F		GGGCTGTCAGATGAACGAG			
pefA	*pefA*	TCACTGTCTCCTGGGCTTCT	219	50°C	This study
pefA		CTTCAGTCTGGCCACCTTTC			
potE-R	*potE*	AATTCAAAACGCGGTGAGAC	1319	55°C	This study
potE-F		TGTCGTGCAGCTCACAATTC			
rep1IF	*repI*	CGAAAGCCGGACGGCAGAA	139	50°C	[17]
rep1IR		TCGTCGTTCCGCCAAGTTCGT			
repA/CF	*repA*	GAGAACCAAAGACAAAGACCTGGA	465	50°C	[17]
repA/CR		ACGACAAACCTGAATTGCCTCCTT			
spvC1	*spv*	AACTCCTTGCACAACCAAATG	230	50°C	This study
spvC1		ACCATATCCCTGAGCACACTG			
strAF	*strA*	AGCAGAGCGCGCCTTCGCTC	684	61°C	[25]
strAR		CCAAAGCCCACTTCACCGAC			
sul1F	*sul1*	TCACCGAGGACTCCTTCTTC	631	60°C	[25]
sul1R		AATATCGGGATAGAGCGCAG			
tetAF	*tetA*	GCTACATCCTGCTTGCCTTC	210	70°C	[28]
tetAR		CATAGATCGCCGTGAAGAGG			
tetBF	*tetB*	TTGGTTAGGGGCAAGTTTTG	659	64°C	[28]
tetBR		TTGGTTAGGGGCAAGTTTTG			
tetGF	*tetG*	CCGGTCTTATGGGTGCTCTA	693	56°C	[25]
tetGR		CCAGAAGAACGAAGCCAGTC			
thdFF	*thdF*	TTGATTTTCCGGATGAGGAG	1553	55°C	This study
thdFR		AGGCATTGACAAAGGTCAGG			

^a ^3' CS; 3' conserved segment of class I integrons, 5' CS; 5' conserved segment of class I integrons. Primers intAF2a, intAF2b, intAF3, intAF4, intAR3 and intAR4 were designed to sequence the intAF/intAR amplicon.

**Figure 2:**
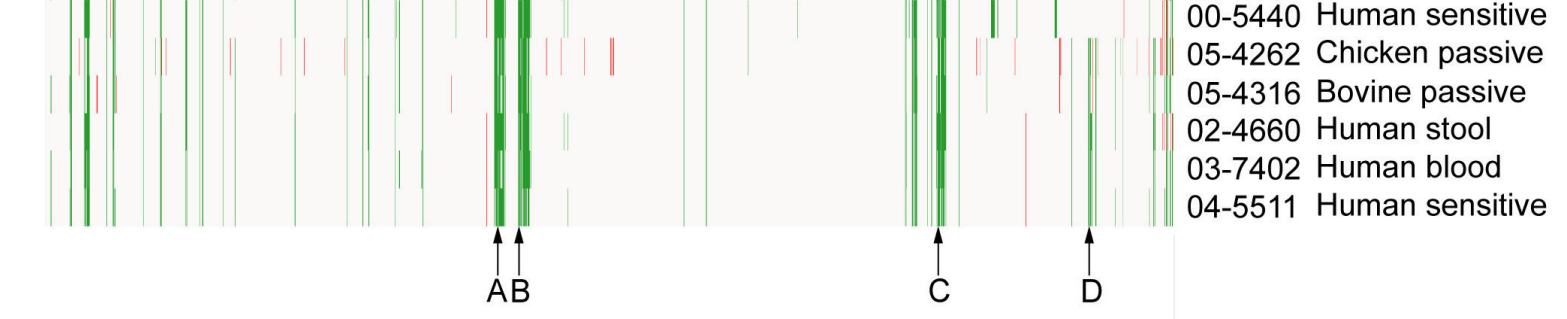
**DNA microarray-based comparative genomics of *S*. Heidelberg.** Array probes represent the linear order of *S*. Typhimurium LT2 coding sequences from left to right, and with the *Salmonella *genomic island 1 (SGI1) present at the far-left side. White denotes similarity to LT2, green denotes putative divergence and red represents putative duplication or copy number change. The region STM0691–0704 which was putatively divergent between *S*. Heidelberg strains is represented by "A". Clusters of bacteriophage-related determinants that are divergent in *S*. Heidelberg compared to *S*. Typhimurium: B, STM0892–0929 (Fels-1 prophage); C, STM2584–2636 (Gifsy-1 prophage); D, STM2694–2739 (Fels-2 prophage).

Comparative genomic hybridization (CGH) between *S*. Heidelberg genomic preparations (Table 1) and a DNA microarray based upon *S*. Typhimurium LT2 was used to attempt to identify variable regions or traits between *S*. Heidelberg strains that were not detected by either PFGE or SBT. The CGH data indicated that there were several bacteriophage-related determinants that were putatively divergent or absent amongst all of the *S*. Heidelberg isolates in reference to *S*. Typhimurium LT2 (Figure 2). These included the Fels-1, Fels-2 and Gifsy-1 prophage genes, which is an observation similar to previous CGH studies that demonstrated variable carriage of prophage genes amongst *Salmonella *serovars [12-14]. Comparison of the CGH patterns between *S*. Heidelberg isolates identified limited strain-to-strain variation between isolates, with the exception of STM0691–STM0709 (Figure 2). The *fldA*, *miaB*, *potE *and *furR *loci encoded in this putatively divergent region were amplified and sequenced from 5 *S*. Heidelberg isolates (05–1147, 00–5440, 03–7402, 05–4262 and 02–4660) however, no nucleotide polymorphisms were observed throughout these gene sequences (data not shown). These data indicated that the minimal strain-to-strain differences identified by CGH may actually be due to experimental variation rather than actual biological differences. In addition, the limited genomic diversity between *S*. Heidelberg isolates identified by this microarray-based method may be representative of the true level of diversity, however, the lack of *S*. Heidelberg specific sequences on the array may have prevented the ability to distinguish between isolates. In the absence of a sequenced *S*. Heidelberg genome, CGH studies based on a *S*. Heidelberg-specific microarray platform are not currently possible.

### Resistance Determinants

Resistance phenotypes were determined by minimum inhibitory concentration [10] testing (Table 1). The isolates were then screened by PCR to determine the corresponding genotypes and to examine whether the resistance genotypes of isolates could be indicative of the isolate source (Table 1; Table 2). All *S*. Heidelberg isolates that exhibited an ESC resistance phenotype were PCR positive for the *bla*_cmy-2 _gene, with the exception of a single bovine isolate (05–4260). The *bla*_cmy-2 _gene was also responsible for the ESC resistance phenotype of five *E. coli *isolates included in the study to represent other sources of ESC resistance determinants disseminating in the agri-food continuum along with *S*. Heidelberg. The DNA sequence of the *bla*_cmy-2 _gene was identical amongst all of the *S*. Heidelberg isolates and the five *E. coli*.

Tetracycline resistance was mediated by a combination of *tetA *and *tetB *genes in *S*. Heidelberg and solely *tetA *in *E. coli *(Table 1). Chloramphenicol resistance was mediated by *aadB*, *cmlA*, *floR *and streptomycin resistance was mediated by *strA *alone or in combination with either *aadA1 *or *aadA2 *or both. Trimethoprim resistance was mediated by the *dhfRA1 *gene. Sulfizoxazole/sulfamethoxazole resistance was mediated by the *sul*1 gene in 6 of 9 *S*. Heidelberg isolates. The gene mediating sulfizoxazole/sulfamethoxazole resistance in the remaining 3 isolates was not identified. The carriage of the *sul*1 genes by isolates indicated the possible presence of class I integrons [15]. Accordingly, class I integrons could be amplified from 5 *S*. Heidelberg and 3 *E. coli *isolates (Table 3). The *S*. Heidelberg integron amplicons were either 1.2 or 3.0 kbp and DNA sequencing identified several resistance determinants carried within the variable region of the integrons in addition to *sul*1 carried within the 3' conserved segment (Table 3). The integron amplified from strain 02–5785 could not be successfully sequenced. Identical integrons encoding the *dhfRA1 *trimethoprim resistance determinant were amplified from 02–4660 and 05–4264. Three *E. coli *isolates carried identical class I integrons encoding *aadA1*. Notably, no class I integrons were amplified from chicken agri-food isolates indicating that these isolates have acquired resistance determinants by other mechanisms.

**Table 3:** Characterization of class I integrons encoded by *S*. Heidelberg and *E. coli*. Integrons were amplified using primers intAF and intAR [25] and sequenced using these same primers, and if necessary to achieve complete coverage, intAF2a, intAF2b, intAF3, intAF4, intAR3 and intAR4 (Table 2).

Isolate	Source	Integron length (kbp)	Location	Genes encoded^a^
01–7169	Human – stool	1.2	Unknown ^b^	*aadA2*
02–4660	Human – stool	1.2	HMW plasmid	*dhfRA1*
02–5785	Human – stool	1.2	Unknown ^c^	nd
03–0845	Human – stool	3.0	HMW plasmid	*aadB, cmlA*
05–4264	Bovine – passive	1.2	Unknown ^c^	*dhfRA1*
*E. coli *830	Chicken – abattoir	1.0	HMW plasmid	*aadA1*
*E. coli *831	Chicken – abattoir	1.0	HMW plasmid	*aadA1*
*E. coli *833	Chicken – abattoir	1.0	HMW plasmid	*aadA1*

^a ^*aadA1*, streptomycin adenyltransferase; *aadA2*, streptomycin/spectinomycin adenyltransferase; *aadB*, aminoglycoside adenyltransferase; *cmlA*, chloramphenicol/florphenicol efflux; *dhfrA1*, dihydrofolate reductase; nd, not determined

^b ^no HMW plasmids were observed in this strain

^c ^HMW plasmids were observed, but could not be isolated from this strain after transformation attempts

### Genetic Variation Attributable to the carriage of Plasmids

The genetic diversity between isolates examined by PFGE, SBT and CGH was limited at chromosomal determinants thus no genetic traits suitable for use in source tracking could be identified. However, different resistance determinants and integrons were observed therefore the plasmid content of the isolates was determined to examine another possible source of genetic variation. The total plasmid content amongst sensitive isolates and isolates resistant to one or more antimicrobial varied due to the carriage of two high molecular weight (HMW) plasmids: one common to all strains and a larger plasmid present only in ESC resistant strains. The common plasmid may represent the HMW virulence plasmid that has been described in other *S. enterica *subsp *enterica *serovars [16], however, the virulence determinants *spv*, *pefA *and *rck *were not detected in the *S*. Heidelberg strains by PCR (data not shown). One or more low molecular weight (LMW) plasmids ranging from 3 to 6 kbp were also observed in each strain regardless of resistance phenotype (data not shown).

**Figure 3:**
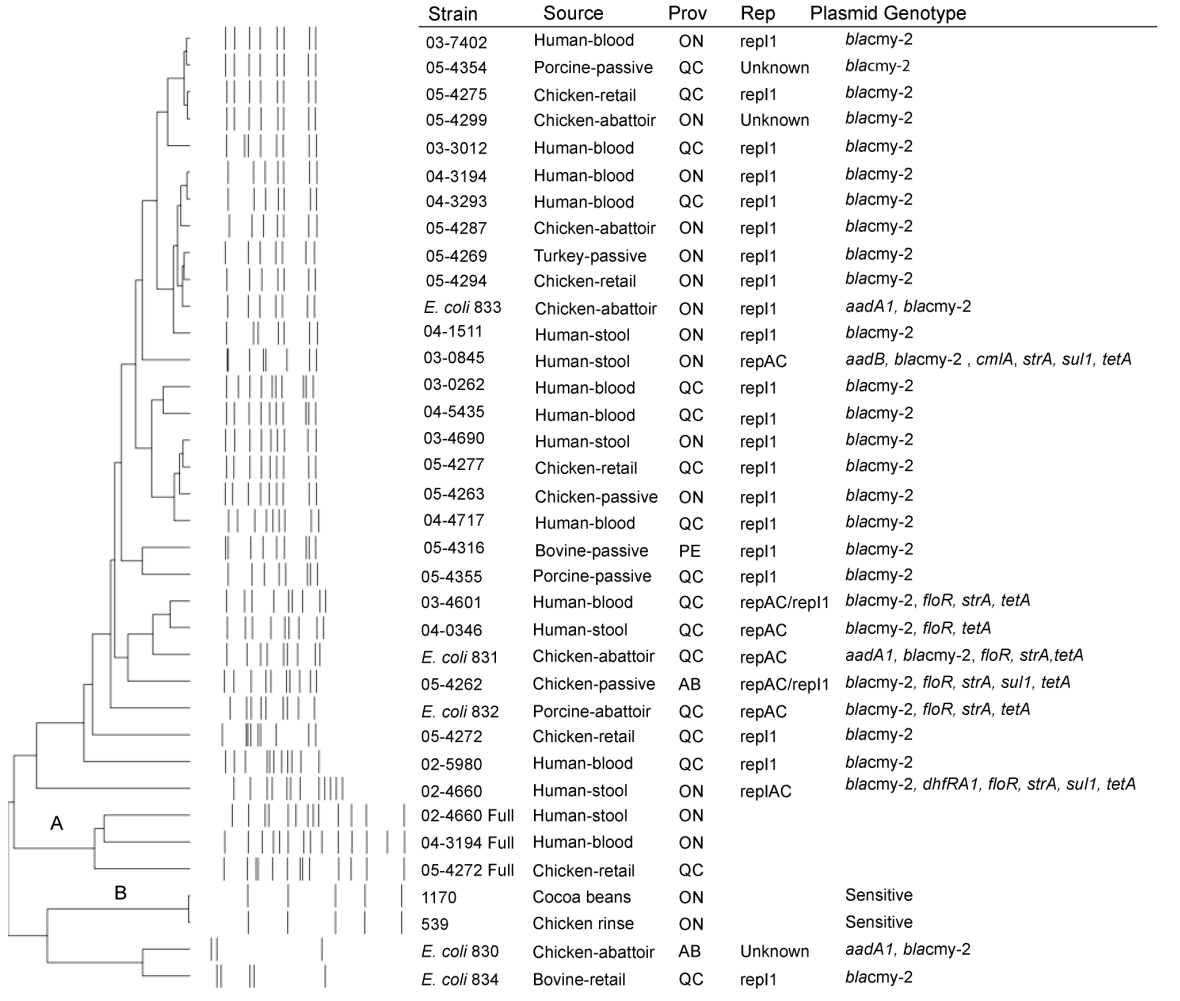
**RFLP of the *bla*_cmy-2 _plasmid using *Bgl*II. Dendrogram created with Bionumerics version 4.0 using UPGMA with a fuzzy band coefficient of correlation, 2% optimization and 10% tolerance.A **– indicates RFLP performed on the full plasmid profile isolated from resistant *S*. Heidelberg strains. **B **– indicates RFLP performed on the full plasmid profile from sensitive *S*. Heidelberg strains. No scale bar is reported due to the high band tolerance settings used in this analysis.

Isolation of plasmids mediating cephalosporin resistance was achieved by electroporating the total plasmid preparation from individual ESC resistant isolates into One Shot^® ^TOP10 Electrocomp™ *E. coli *cells. Transformant colonies containing ESC resistance determinants were selected with cefoxitin and the *bla*_cmy-2 _gene was amplified by PCR from all of the transformant strains. Transformant colonies could not be obtained using plasmid DNA preparations from strains 02–5785 and 05–4264. Of the two HMW plasmids originally observed in the *S*. Heidelberg strains, only the larger plasmid was isolated from all cefoxitin resistant transformants. Restriction fragment length polymorphism (RFLP) analyses were performed on the resistance plasmids using *Bgl*II to determine genetic relatedness (Figure 3). The presence of the *bla*_cmy-2 _gene on this HMW plasmid (hereafter referred to as the *bla*_cmy-2 _plasmid) was confirmed by Southern blot of the RFLP fragments. The *bla*_cmy-2 _gene could be localized to the largest RFLP (~20 kbp) fragment in all isolates with the exception of two human blood isolates (03–3012 and 02–5980; data not shown). The 1.2 kbp and 3.0 kbp integrons encoded by isolates 02–4660 and 03–0845 respectively, were localized to the *bla*_cmy-2 _HMW plasmid by screening the *bla*_cmy-2 _transformant colonies by PCR and by Southern blot of the RFLP fragments (Table 3). The integron amplified from isolates 05–4264, 01–7169 and 02–5785 could not be localized to the *bla*_cmy-2 _HMW plasmid because the plasmid could not successfully be isolated from these strains or they did not harbour HMW plasmids (Table 3).

The *bla*_cmy-2 _plasmids extracted from *E. coli *transformants exhibited diverse RFLP patterns (Figure 3) suggesting that multiple plasmid backbones were involved in the emergence of ESC in *S*. Heidelberg. Notably, isolates from different sources (i.e. human clinical and porcine clinical; human clinical and chicken clinical or retail) had identical RFLP patterns. Identical RFLP patterns were also obtained from *S*. Heidelberg 05–4294 and *E. coli *833 indicating that *bla*_cmy-2 _plasmids are successfully transferred between species. The horizontal transfer of *bla*_cmy-2 _plasmids between bacterial species and the transfer of the *bla*_cmy-2 _gene between plasmid backbones limits the utility of plasmid characterizations to discern the epidemiologic relationships amongst isolates. RFLP analyses were also performed on the full plasmid preparations from *S*. Heidelberg sensitive and resistant *S*. Heidelberg isolates to characterize the HMW plasmid common to all *S*. Heidelberg isolates (Figure 3). Restriction fragments of the common plasmid in the sensitive strains corresponded to fragments in the resistant strains indicating that these plasmids are similar between isolates.

Replicon typing schemes have recently been hypothesized to be a more accurate method of determining plasmid relationships than RFLP [17]. We investigated the ability of a PCR-based replicon typing scheme to supplement plasmid RFLP data. Two different replicons, *repA/C *and *repI1*, were identified for the *bla*_cmy-2 _plasmids by screening the *bla*_cmy-2 _transformants (Table 1). These plasmids were also screened by PCR and Southern blot for individual resistance determinants. All 7 *bla*_cmy-2 _plasmids isolated from 5 *S*. Heidelberg and 2 *E. coli *that encoded a *repA/C *replicon also encoded multi drug resistance (combinations of *aadA1, dhfRA1, floR, strA, sul1*, and *tetA*) (Table 1, Figure 3). The presence of *repA/C bla*_cmy-2 _plasmids that carry resistance cassettes have also been identified amongst *S*. Typhimurium isolates [18]. The class I integrons localized to the *bla*_cmy-2 _plasmid demonstrated a mechanism for the generation of MDR. The *repA/C *plasmids had diverse RFLP patterns compared to the *repI1 *plasmids further indicating the mosaic nature of these plasmids. The *repA/C *were unable to be transferred by conjugation to the recipient *E. coli *strain RG192 with the exception of 03–7402 that was conjugative.

The majority of the *bla*_cmy-2 _plasmids isolated from both *S*. Heidelberg and *E. coli *were shown to carry *repI1 *replicons and only the *bla*_cmy-2 _resistance gene was detected by PCR and Southern blot (Table 1, Figure 3). All *repI1 *plasmids tested successfully transferred by conjugation to a recipient *E. coli *strain with the exception of 04–3293. The conjugative ability of *repI1 *plasmids can help explain their widespread presence amongst the *S*. Heidelberg isolates. A single chicken-passive isolate was shown to carry both the *repA/C *and *repI1 *replicons along with the *bla*_cmy-2_, *strA*, and *floR *resistance determinants. Three plasmids encoding only the *bla*_cmy-2 _resistance determinant did not encode either the *repA/C *or *repI1 *replicons. The *repA/C *MDR plasmids were isolated from human-clinical and both chicken and porcine samples, and similarly, the *repI1 *plasmids were isolated from diverse sources, therefore replicon typing could not be used to establish a link between human illness and a particular agri-food *S*. Heidelberg source.

## Conclusion

There was limited genetic diversity at the chromosomal level amongst the *S*. Heidelberg isolates. Plasmid content exhibited strain-to-strain variation and was responsible for differences in antimicrobial susceptibility between isolates. No suitable genetic trait was identified to permit source tracking of human S. Heidelberg infections. However, the paucity of *S*. Heidelberg in other agri-food sources such as pigs or cows indicates that chicken agri-food products are the most likely source of human salmonellosis among the commodities surveyed by CIPARS [8]. *S*. Heidelberg is also frequent in clinical turkey samples [8], but CIPARS does not currently routinely survey *Salmonella *in turkey at the abattoir or retail level and the frequency of *S*. Heidelberg in healthy turkey is unknown. It is therefore possible that if the main reservoir for *S*. Heidelberg is poultry, amongst this population of strains there is in fact limited overall genetic diversity and no experimental method will identify epidemiologically significant genetic traits.

In addition, this study demonstrates the elements responsible for ESC resistance and MDR in *S*. Heidelberg. ESC resistance of *S*. Heidelberg isolates could be attributed to the carriage of *bla*_cmy-2 _on HMW resistance plasmids and other resistance determinants were localized to integrons. Two resistance plasmids were observed: *repA/C *non-conjugative HMW plasmids encoding ESC resistance in combination with other resistance determinants, and conjugative *repI1 *plasmids encoded solely ESC resistance.

## Methods

### Bacterial strains

39 *S*. Heidelberg and 5 *Escherichia coli *isolates included in this study were obtained from human clinical, retail, and agri-food animal sources by CIPARS (Table 1). Antibiotic susceptible *S*. Heidelberg isolates 1170, 539 and 564 were provided by the Bureau of Microbial Hazards. Phage typing was performed as previously described [19].

### Antimicrobial Susceptibility Testing

Resistance to antimicrobials was determined at the Laboratory for Foodborne Zoonoses (LFZ) or the National Microbiology Laboratory (NML) by broth microdilution using the Sensititre™ ARIS Automated Microbiology System (Trek Diagnostic System Ltd, Cleveland Ohio). Breakpoints for resistance were taken from the NCCLS/CLSI guidelines and were as follows: amoxicillin-clavulanic acid; ≥ 32/16 μg/ml, ampicillin; ≥ 32 μg/ml, cefoxitin; ≥ 32 μg/ml, ceftriaxone; ≥ 64 μg/ml, cephalothin; ≥ 32 μg/ml, chloramphenicol; ≥ 32 μg/ml, ciprofloxacin; ≥ 4 μg/ml, gentamicin; ≥ 16 μg/ml, kanamycin; ≥ 64 μg/ml, nalidixic acid; ≥ 32 μg/ml, sulfizoxazole/sulfamethoxazole; ≥ 512 μg/ml, tetracycline; ≥ 16 μg/ml, trimethoprim-sulfamethoxazole; ≥ 4/76 μg/mL [20] and ceftiofur; ≥ 8 μg/ml [21]. The breakpoint used for streptomycin resistance was ≥ 64 μg/ml [22]

### Pulsed-field Gel Electrophoresis (PFGE)

PFGE was performed by PulseNet Canada at the NML according to the PulseNet USA protocol using *Xba*I and *Bln*I [23]. Pattern analysis and dendrogram construction were performed using the BioNumerics version 4.0 software package (Applied Maths, Austin, TX) with 1% tolerance and 1.5% optimization.

### Genomic DNA extractions

Wild type *S*. Heidelberg or *E. coli *strains were used to inoculate 4 ml of Luria Bertani [17] broth (Invitrogen, Carlsbad, CA). Alternatively, One Shot^® ^TOP10 Electrocomp™ (Invitrogen) *E. coli *transformants containing *S*. Heidelberg *bla*_cmy-2 _plasmids were inoculated into 6 ml LB broth containing 20 μg/μl cefoxitin (Sigma-Aldrich, Oakville, ON). Cultures were incubated overnight at 37°C with rotation at 200 rpm. Bacterial cells were collected by centrifugation for 5 min at 5000 rpm and resuspended in 2 ml TE buffer (Sigma-Aldrich) (10 mM Tris-HCL, 1 mM EDTA, pH 8.0). Lysozyme (Roche Diagnostics, Indianapolis, IN) (0.5 mg/ml), RNase (Roche Diagnostics) (1.5 μg/ml), and proteinase K (10 mM Tris-HCL pH 7.5, 20 mM CaCl_2_, 50% glycerol) (Sigma-Aldrich) (0.12 mg/ml) were added to the cell resuspension mixture. Following incubation at 37°C for 1 h, sodium dodecyl sulphate (SDS) (Ambion, Austin, TX) was added to a concentration of 0.1% (wt/vol) and the mixture was incubated at 65°C until clearing occurred. The mixture was then transferred to Phase Lock Light tubes (Eppendorf, Hamburg Germany) for phenol-chloroform DNA extraction using a volume of phenol-chloroform:isoamyl alcohol (25:24:1) (Invitrogen) equal to that of the cell resuspension mixture. Phenol-chloroform:isoamyl alcohol extraction was repeated until the aqueous layer was clear. Following a final extraction with an equal volume of chloroform (Fisher, Ottawa, ON), the aqueous layer was transferred to a new tube. DNA was precipitated at -20°C for 20 min using 0.6 vol of isopropanol (Fisher) and 0.1 vol 3 M sodium acetate (pH 5.5) (Ambion). Following precipitation, DNA was washed with 70% ethanol and resuspended in 200 μl TE buffer. DNA was quantified on a NanoDrop ND-1000 (NanoDrop Technologies, Rockland, DE) and diluted to 20 ng/μl for use as PCR template.

### PCR and DNA Sequencing

PCR reactions for *tetA*, *tetB *and *tetG *were performed using 1 unit of Fast start *Taq *DNA polymerase (Roche Diagnostics) in a reaction mixture containing 1 × Fast start *Taq *DNA polymerase buffer with MgCl_2 _(Roche Diagnostics), 1 mM MgCl_2 _(Roche Diagnostics), 0.2 μM dNTP mixture (Invitrogen), 0.2 μM each primer (Table 2), 20 ng template DNA and distilled water DNAse, RNAase free (Invitrogen) to 25 μl. The thermocycling parameters used for *tetA*, *tetB *and *tetG *reactions containing Fast start Taq DNA polymerase were: initial denaturation at 94°C for 5 min followed by 35 cycles of denaturation at 94°C for 30 sec, annealing at a primer specific temperature listed in Table 2 for 30 sec, extension at 72°C for 1 min followed by one final extension at 72°C for 7 min. All other PCR reactions were performed using 1 unit of Platinum Hifi *Taq *DNA Polymerase High Fidelity (Invitrogen) in a reaction mixture containing 1 × High Fidelity PCR Buffer (600 mM Tris-SO_4 _[pH 8.9], 180 mM ammonium sulfate) (Invitrogen), 0.2 mM dNTP mixture (Invitrogen), 2 mM MgSO_4 _(Invitrogen), 0.3 μM each primer (Table 2), 20 ng template DNA and distilled water DNAase, RNAase free (Invitrogen) to 25 μl total volume. The thermocycling parameters used for *intA*, *potE *and *miaB *PCR reactions containing Platinum Hifi *Taq *were: initial denaturation at 94°C for 5 min followed by thirty cycles of denaturation at 94°C for 30 sec, annealing for 30 sec, at a primer specific temperature listed in Table 2, extension for 60 sec (*intA*) or 90 sec (*potE, miaB*) at 68°C followed by one final extension at 68°C for 7 min. The thermocycling parameters used for all other reactions containing Platinum Hifi *Taq *were: initial denaturation at 94°C for 5 min followed by 30 cycles of denaturation at 94°C for 30 sec, annealing for 30 sec, at a primer specific temperature listed in Table 2, extension for 30 sec at 68°C followed by one final extension at 68°C for 7 min. PCR amplicons were resolved by agarose gel electrophoresis on 1.5% agarose gels in 0.5× TBE buffer (Sigma-Aldrich) at 120 V for 60 min. Sequencing was conducted by the DNA Core facility at the NML using an ABI3730 apparatus (Applied Biosystems, Foster City, CA) with the primers used to generate the template (Table 2). Complete sequencing of the *bla*_*cmy*-2 _PCR product required the design of additional sequencing primers listed in Table 2.

### Comparative Genomic Hybridization

DNA microarrays were constructed as previously described using 4492 commercially supplied 70-mer oligonucleotides (Qiagen, Mississauga, ON) representing the coding sequences of the *S*. Typhimurium LT2 genome as well as all putative open reading frames from *Salmonella *Genomic Island I (SGI1) [24]. Genomic DNA from test *S*. Heidelberg and reference *S*. Typhimuirum LT2 strains was isolated by phenol-chloroform extraction, sheared by nebulization and labelled by Cy3 dCTP or Cy5 dCTP incorporation as previously described [19]. Labelled DNA from test and reference strains was hybridized to the array as previously described with each test-versus-reference comparison performed in triplicate and at least one of the slides hybridized as a dye swap. Following hybridization, slides were sequentially washed in buffer 1 (1 × SSC [3.0 M sodium chloride, 0.3 M sodium citrate] and 0.2 % SDS) for 6 min at 56°C, buffer 2 (0.1 × SSC and 0.2 % SDS) for 4 min at room temperature, and twice in buffer 3 (0.1 × SSC) for 2 min at room temperature. Slides were scanned using an Agilent DNA microarray scanner (Agilent Technologies, Mississauga, ON). Data analysis was conducted as previously described to identify specific loci that were absent or divergent between different *S*. Heidelberg strains [19].

### DNA sequence-based typing (SBT)

SBT was conducted using a previously published scheme based on the *fimA, manB*, and *mdh *loci [11]. PCR and sequencing were conducted using primers and annealing temperatures listed in Table 2. Sequence analysis was carried out using Seqman II (DNAstar Inc) and sequences were concatenated to create allelic profiles for each strain.

### Plasmid Profiles

A single colony was used to inoculate 8 ml of LB broth and incubated overnight at 37°C with rotation at 200 rpm. Complete plasmid profiles were isolated from 2 ml of overnight culture using a QIAGEN plasmid mini kit (Qiagen) according to manufacturer's directions. Plasmids were resuspended in 25 μl TE buffer (10 μM Tris-HCL pH 8.0, 1 μM EDTA) (Sigma-Aldrich) and resolved by gel electrophoresis on 0.9 % agarose gels in 0.5 × TBE buffer (Sigma-Aldrich) at 90 V for 90 min. Following staining for 20 min in ethidium bromide (2 μg/ml) and destaining for 20 min in ddH_2_O gels were visualized by UV transillumination using a BioRad GelDoc XR (Bio-rad). Plasmid size was estimated using either a supercoiled DNA ladder (Invitrogen) containing supercoiled plasmids ranging from 2 to 16 kbp.

### Electroporation

Transformation of the plasmids encoding *bla*_cmy-2 _was achieved by adding 5 μl of the plasmid preparations to 50 μl OneShot^® ^TOP10 Electrocomp™ *E. coli *cells (Invitrogen) in a 0.1 cm chilled cuvette (Cell Projects, Kent, UK). A BioRad Gene Pulser (Bio-Rad) was used to apply a 1.25 kV pulse and 1 ml of S.O.C medium (Invitrogen) was immediately added to the cuvette and the contents transferred to a sterile culture tube. Following incubation of the transformation culture at 37°C for 60 min, 20 and 200 μl aliquots were plated onto LB agar (Invitrogen) plates containing 20 μg/ml cefoxitin (Sigma-Aldrich) and incubated overnight at 37°C. Potential transformant colonies were inoculated into 8 ml LB broth containing 20 μg/ml cefoxitin and incubated overnight at 37°C with agitation. DNA extractions were then conducted and PCR using the *bla*_cmy-2 _primer set was performed to verify transformants carried the *bla*_cmy-2 _gene.

### Plasmid isolation from transformants

A single transformant colony was inoculated into 8 ml of LB broth with 20 μg/ml cefoxitin and incubated overnight at 37°C with rotation at 200 rpm to create a starter culture. Starter culture was diluted 1/500 into 150 ml LB broth with 20 μg/ml cefoxitin and grown overnight at 37°C with rotation at 200 rpm. Plasmids were isolated from 50 ml of overnight culture using a Qiagen plasmid midi kit according to the manufacturer's directions with the following modification: plasmid DNA was precipitated using 2 ml 7.5 M ammonium acetate (Sigma-Aldrich) in combination with 0.7 vol of isopropanol (Fisher) and resuspended in 100 μl TE buffer.

### Restriction Fragment Length Polymorphism (RFLP)

Purified plasmid DNA (25 μl) was digested overnight at 37°C with 20 units of *Bgl*II (New England Biolabs, Pickering, ON). The resulting plasmid fragments were separated by gel electrophoresis on 0.7 % Tris-acetate-EDTA (TAE) (Gibco) agarose gels at 60 V for 6 h in TAE (400 mM Tris-acetate, 10 mM EDTA). A 1 Kb Plus DNA Ladder (Invitrogen) and Track it λ DNA/*Hind *III fragments (Invitrogen) were used as molecular size standards. Gels were stained with ethidium bromide (2 μg/ml), destained overnight in ddH_2_O at 4°C, visualised with UV transillumination and photographed using a Bio-Rad Gel Doc XR. RFLP pattern analysis and was conducted using Bionumerics version 4.0 software with 2% optimization and 10% tolerance. A dendrogram based on RFLP patterns was generated in Bionumerics using the unweighted pair group method of analysis (UPGMA) with a fuzzy band logic coefficient of correlation, 2% optimization, and 10% tolerance.

### Southern Blot

RFLP gels were depurinated in 250 mM HCI for 12 min, denatured in a 1.5 M NaCl, 0.5 M NaOH solution for 30 min and finally neutralized in a 1.5 M NaCl, 0.5 M Tris-HCl pH 7.5 solution for 30 min. DNA was transferred by capillary blotting to a positively charged nylon Hybond-N+ (Amersham Biosciences, Little Chalfont, UK) membrane using the TurboBlotter system (Schleicher & Schuell, Keene, NH) according to manufacturer's directions with 10 × SSC (Ambion) transfer buffer. Membranes were rinsed in 6 × SSC (Ambion) and DNA was fixed to the membrane by UV treatment. Nucleic acid labelling and detection was carried out following the manufacturer's directions using the ECL Direct Nucleic Acid Labelling and Detection System (Amersham Life Sciences, Little Chalfont, UK). Labelled probes targeting the *aadA1*, *bla*_*cmy*-2_*, dhfRA1, floR, strA, sul1, tetA, and tetB *genes were generated from PCR products. PCR amplicons were purified using the QIAquick PCR Purification Kit according to manufacturer's directions, quantified on a NanoDrop ND-1000 and diluted to 10 ng/μl. Amplicons (150 ng) were denatured by boiling for 5 min and snap cooling on ice for 5 min. Probe DNA was labelled at 37°C for 10 min through the addition of equal volumes of DNA labelling reagent (Amersham Life Sciences) and gluteraldehyde (Amersham Life Sciences). Membranes were pre-hybridized at 42°C for 30 min in 25 ml pre-heated ECL gold hybridization buffer (Amersham Life Sciences) containing 0.5 M NaCl and 5 % (w/v) blocking agent (Amersham Life Sciences). Labelled probe was added to hybridization buffer and hybridization was allowed to proceed overnight at 42°C in a Fisher Isotemp hybridization oven (Fisher). Following hybridization, excess probe was removed by washing twice with primary wash buffer (0.5 × SSC, 0.4 % SDS) at 42°C for 20 min and twice with 2 × SSC for 5 min at room temperature. The presence of target gene was detected on Hyperfilm ECL (Amersham) autoradiography film according to manufacturer's directions. Film was developed in a Feline™ developer (Fisher).

### Conjugation

*Salmonella *Heidelberg strains were used as *bla*_cmy-2 _plasmid donor strains. Recipient *E. coli *RG192 was serially passaged against rifampicin (Sigma-Aldrich) until resistance to 384 μg/ml was achieved. Cefoxitin (20 μg/ml) and rifampicin (384 μg/ml) were used as selective agents for the donor and recipient strains, respectively. Single colonies of donor and recipient strains from selective plates were inoculated into 8 ml of LB broth containing the appropriate selective antibiotic and grown overnight at 37°C at 200 rpm. Overnight cultures were then sub-cultured into LB broth without selective antibiotic and incubated at 37°C for 5 h at 200 rpm. Recipient and donor cells were combined in a 4:1 ratio in LB broth. Following overnight incubation at 37°C, transconjugants were selected by plating onto LB agar containing cefoxitin (20 μg/ml) and rifampicin (384 μg/ml). The transfer of *bla*_cmy-2 _was confirmed by performing PCR to detect the presence of the *bla*_cmy-2 _gene followed by isolation of plasmids from *E. coli *transconjugants.

## Abbreviations

Canadian Integrated Program for Antimicrobial Resistance Surveillance [8]; comparative genomic hybridization (CGH); extended spectrum cephalosporin (ESC); minimum inhibitory concentration [10]; multi-drug resistant (MDR); National Microbiology Laboratory (NML); pulsed-field gel electrophoresis (PFGE); restriction fragment length polymorphism (RFLP); sequence-based typing (SBT).

## Authors' contributions

AKA performed resistance gene PCR, CGH, SBT and plasmid analyses for this study, as well as drafted the manuscript. ABO and DMT participated in CGH, plasmid analyses and assisted in drafting and revising the manuscript. L–KN, KD, RI and CIPARS assisted in conceiving the study, provided background and epidemiological data for CIPARS strains and revised the manuscript. MWG supervised the project and drafted and revised the manuscript. All authors read and approved the final manuscript.
